# Outsourcing Swimming Education—Experiences and Challenges

**DOI:** 10.3390/ijerph18010006

**Published:** 2020-12-22

**Authors:** Bjørn Harald Olstad, Pernille Ravn Berg, Per-Ludvik Kjendlie

**Affiliations:** Department of Physical Performance, Norwegian School of Sport Sciences, 0863 Oslo, Norway; pernille-ravn-berg@hotmail.com (P.R.B.); Per-Ludvik.Kjendlie@usn.no (P.-L.K.)

**Keywords:** external providers, physical education, swimming abilities, elementary school, pedagogy

## Abstract

In Norway, swimming and lifesaving education (swimming education) is an obligatory part of physical education, with explicit learning aims after grade four. After recent reports of Norwegian pupils achieving low scores in swimming abilities, the Government has outlined strategies for improving swimming education. There is a notable trend toward using external providers in delivering swimming education. This article examines the outsourcing of swimming education in Norwegian primary schools. Eighteen semi-structured interviews were conducted with school leaders, physical education teachers and swimming instructors involved in outsourcing arrangements. The outsourcing was organized through private providers, municipalities, or local swimming clubs. Data were analyzed thematically and separated into highlighted areas of outsourcing practices. The results showed that outsourcing may be a solution for schools that lack staff with swimming experience and knowledge. It also indicates that teacher courses, professional development through collaboration, and strategies for measuring quality would improve swimming education.

## 1. Introduction

Norway is a country wrapped in sea, divided by fjords, and filled with lakes and rivers. Each year a relatively high number of people drown: In 2017, 2018, and 2019, the numbers were 94, 102, and 86, respectively [1]. This is a rate of 1.8 drownings per 100,000 inhabitants. Swimming education in school is considered one way to prevent drownings, and of allowing pupils to discover the joy and freedom of good swimming abilities [2]. Swimming abilities can be defined as being able to move through the water with effective propulsion, enjoying the water, and mastering a variety of skills, both over and under the water [3]. During the 1980s, there was a mutual agreement between the Norwegian School of Sport Sciences, the Norwegian Swimming Federation and the Norwegian Lifesaving Society that there are eight basic skills that need to be mastered in order to be able to swim [4], as outlined in research by Stallman et al. [5]. In 1996, the Nordic Countries defined ‘swimming ability’ as a term applicable to a person who can fall into water with the head submersed, regain contact with the surface, and thereafter swim continuously for 200 m, of which at least 50 m is on the back [6]. According to Stallman et al. [5], varied competence in water is equally as important as technical skill training, and this competence can prevent drowning. These authors believe that swimming skills are only one part of “water safety” and that both knowledge and attitudes must be part of any comprehensive program [5]. The same view is seen in the idea of “aquatic readiness”, representing an individual’s unique set of aquatic experiences, which predict the most likely task a swimmer may be ready to acquire next in any aquatic environment [7]. However, the findings from one study on children and their parents’ conceptions imply that it is normal to think of swimming ability as just achieving propulsion in the water [8].

The Norwegian Swimming Federation, Norwegian School of Sport Sciences, Norwegian Life Saving Society, and others collaborate closely with the Department of Education in developing swimming education and curriculum for schools. The curriculum for physical education has competence aims after grade four, which were revised in 2015 by the Department of Education [2]. The Norwegian curriculum (revised 2015) states that all children should be proficient in swimming by grade four (9–10 years old), as part of their physical education curriculum. The criteria for swimming proficiency are: Being able to fall into deep water, swim 100 m in the prone position, surface dive and retrieve an object, stop, rest and float for 3 min, then swim 100 m in the supine position and get yourself out of the water [2]. This new set of criteria is a development and includes several aspects of swimming proficiency. However, the status of what children can perform is based on a somewhat older definition of swimming proficiency, which was to cover a distance of 200 m. The question of skills to cover a 200 m distance does not reflect all aspects of water safety and has its roots in the traditional Nordic definition of swimming ability (covering a distance). The curriculum also states that children in grade four should be able to play and demonstrate basic skills in the water, such as diving, gliding, surface diving, and orienting themselves in the water. Knowledge of danger, how to move safely in, on, and around water, is also part of the curriculum. In grades 7 to 10, when the pupils are 12–13 and 15–16 years old, lifesaving skills become part of the curriculum.

In several reports spanning the last two decades (2003, 2009, and 2013), the swimming abilities of Norwegian fifth graders showed that 50% of the pupils could not swim 200 m [9,10,11]. These surveys were based on pupils’ self-reported skills (their beliefs about their skills) and did not examine their real skills. Previous research has identified a tendency to overestimate self-reported skills both in young people and among parents [12,13,14]. It is therefore possible that less than 50% of the pupils where actually able to swim 200 m. In addition, a large proportion of both pupils and teachers reported wanting more time for swimming education. The surveys also found that only half of the fifth-graders who responded had swimming education during primary school. Another survey conducted by Gjerustad et al. [15] included schools’ opinions of swimming classes in Norway. The survey included questions about the teachers’ competence, the level of goal attainment, access to swimming pools, and the number of pupils in the pool at the same time. Like earlier reports, this study indicated that problems with resources and hours available meant that some schools did not deliver the swimming education their pupils needed to achieve the aims of swimming in physical education. Since swimming and lifesaving constitute a compulsory part of the curriculum, Norwegian pupils have the right to receive sufficient swimming education to reach the curriculum aims. Many schools offer only 10 hours of swimming education annually [15], and many schools do not offer swimming and lifesaving in every grade. There is no national standard for the amount of time assigned for swimming education in the curriculum. In other countries, the number of lessons each year varies; for example, a Finnish study reported as few as 1–2 lessons per year in some schools [16], while Icelandic schools provide swimming education every week or at least 20 hours a year throughout all grades [17].

Gjerustad et al. [15] showed that 15% of the schools in Norway used staff from the local swimming pool to teach swimming, and these staff having no connection to the school. In the region of Oslo and Akershus, 45% of the schools used instructors from swimming clubs, private providers, volunteer organizations, or a swimming instructor hired by the municipality. In Norway, schools are free to use the local market when providing resources such as instructors or facilities. This means that outsourcing physical education is a choice [4]. Research on outsourcing physical education is a relatively new field, with few empirical studies published [18]. “Outsourcing occurs when an organization contracts with another organization to provide services or products of a major function or activity” [19] (p. 270), Thus, outsourcing is based on strategic decisions, whereas purchasing is administrative, and defined as the process of acquiring goods and services.

In Norway, the general classroom teacher, with little or no formal competence in the subject, has also historically led physical education or Health, Sport, and Physical Education (HSPE) [20,21,22]. However, today it is common that a combination of general classroom teachers, physical education teachers, and external providers is responsible for HSPE [21]. British studies point out the frequent use of sport coaches as external providers of physical education [21,23,24]. The coaches employed may represent a range of sports, with soccer and dance coaches occasionally mentioned. The use of coaches is justified by their higher levels of specialist knowledge and lower costs to the schools [23].

Studies show that teachers have found classes held by an external provider as positive for their professional development in physical education [18,22,25,26]. Professional development can also be conducted via teacher courses [27], something teachers wish they were offered [28]. A few publications have explored reasons for the collaboration between schools and external providers [18,26,29]. In a collective case study on Australian secondary schools, principals and physical education teachers commonly referred to educational value, human resources, physical resources, and symbolic resources as reasons for outsourcing. The external providers in the same study were motivated by the educational value, income generation, and advertising of their work [29].

Currently, there is little published material on the outsourcing of swimming education and its consequences. Peden et al. [30] (p. 202) found that in Australia “Aquatic activity was outsourced in 88.1% of primary schools surveyed”. In New Zealand, “Many schools have swimming programs provided by external providers” [25] (p. 10). Swim England also has a strong focus on external lesson providers integrated with school swimming for achieving what they call; great school swimming lessons through collaboration, knowledge transfer, progressive lessons, cross-curricular activities, and that most of the time is spent in the water and not on the side [31].

This study aimed to examine outsourcing arrangements of swimming education in Norwegian primary schools and the mechanisms for contracting external providers. Further, the study aimed to investigate the consequences of these arrangements and how swimming education could be improved through semi-structured interviews with school leaders, physical education teachers, and swimming instructors involved in outsourcing arrangements. The hypotheses of the study were that outsourcing arrangements would be used when schools lack internal swimming competence and that physical education teachers could develop their swimming knowledge and benefit from a professional collaboration with external providers.

## 2. Materials and Methods 

### 2.1. Participants

Qualitative methods were used; one-to-one interviews were conducted with 18 informants. The informants were school leaders (SLs) at schools that outsourced swimming education, teachers who had physical education as a subject at schools that outsourced swimming education, and swimming instructors (SIs) who covered swimming education for schools. The argument for interviewing these three groups was the interest in the different perspectives of the groups. Prior to the interviews, the informants received information regarding the study and provided written consent before participating. The sample consisted of three groups of outsourcing models: Private swimming instructors, instructors from a swimming club, and instructors hired by the municipality (Table 1). The study was approved by the National Centre for Research Data (NSD). (The project identification code is 49757). 

### 2.2. Interview Guide

A semi-structured interview guide was developed for all three groups, containing questions about education, sport and physical education, teaching swimming, competence requirements in swimming, outsourcing, competence, pupils, and economy. A pilot interview was carried out with one SL. The interviews were conducted in a suitable office location at the informants’ workplaces. On average, the interviews lasted 38 min, ranging from 32–57 min. Each interview was audio-recorded and transcribed shortly after completion. The data were stored on an external memory disk and were inaccessible between authorized usages.

### 2.3. Thematic Analysis

**Figure 1:**
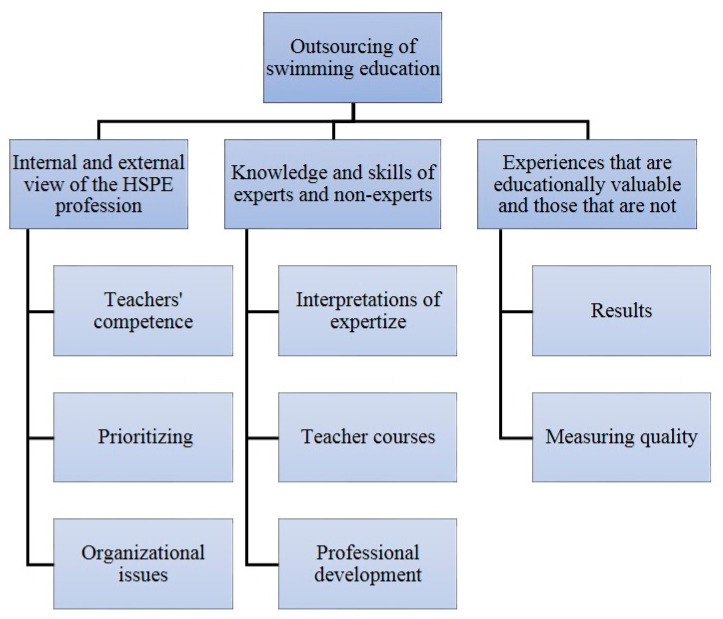
Research aim (1st row), research questions (2nd row), and analytic themes (3rd–5th row). HSPE = Health, Sport, and Physical Education.

The data were analyzed using thematic analysis [32]. This involved examining the data and coding themes and recurring patterns that were of importance to the research questions (Figure 1). MAXQDA (VERBI Gmbh, Berlin, Germany) was used to structure the data. The analysis focused on several aspects of the implications of outsourcing: The internal and the external points of view of the HSPE teaching profession; the knowledge and skills of experts and non-experts; and experiences regarded as educationally valuable and those that were not.

## 3. Results

### 3.1. Teaching Swimming with HSPE Teachers (Internal) or External Providers

#### 3.1.1. Teachers’ Competence

According to statistics, only 48% of physical education teachers in Norwegian primary schools have formal qualifications in physical education (Lagerstrøm, Moafi, and Revold, 2014). In this study, 4/4 physical education teachers, 2/7 SLs, and 3/7 SIs had formal education in physical education, with either physical education teacher education (PETE) or shorter physical education specializations.

The SLs stated that they sent physical education teachers, classroom teachers, assistants, and a combination of these to the swim lessons. A common thing for the SLs was that they were aware of the need to send staff with either knowledge of swimming and/or an interest in participating. All the SLs said that they sent qualified staff with the classes and that they had trained physical education teachers on their staff. Reasons for outsourcing included tradition, educational worth, and organizational issues. Other reasons did not involve shortage of available knowledge. ”We have two physical education teachers, they’re happy they don’t have swimming classes. Neither of them has any particular interest in swimming. If we had a physical education teacher who loved swimming, we might have done something else” (SL 2 and 3). ”We have two teachers with formal physical education and another one who is a trained swimming instructor, but we had to cover the theoretical subjects first and must prioritize using her in the classroom” (SL 1).

#### 3.1.2. Prioritizing

Regardless of the explicit aims after fourth grade, the teachers with competence in swimming are also used for their competence in other subjects. Some of the answers revealed that there were many ways of prioritizing shown by the informants. 

We advise the schools to hire more instructors, but it’s hard. They’re not willing to pay more. If you meet the right person you can find engagement, but we also meet school leaders who don’t want to spend resources on swimming. In general, there is too little knowledge on swimming (SI 5). 

We’re willing to pay the price. Outsourcing saves us time and work and we’re secure having a good offer for the pupils. Our school is large; we had to cover it either way (SL 2 and 3). 

It’s a discussion about key learning areas. We see many things that the children don’t manage yet, maybe some may benefit from using those 20 hours on something other than swimming to have a good life (SL 5).

#### 3.1.3. Organizational Issues

There were differences in the experience SLs and teachers had with swimming education. Several informants commented on the process of outsourcing and a common issue was the lack of information. There were also suggestions on how the organization could improve.

Organizing and planning this have been frustrating. Finding willing staff, courses, time in the pool, and an instructor. There is little information and teaching children to swim is not a focus area in the teacher education programs. It’s a great cost sending one teacher per 15 pupils, and yet we don’t know how much this will cost. We must evaluate later (SL 7). 

We barely knew the resources needed to fulfil this [requirement] and where to get help. When the Government puts out demands, they should offer us swimming instructors, schedule time, and let us cover the extra staff. The extent of organizing swimming education surprised us. In addition to teacher and instructor courses, we need to double the staff, find transportation and it takes minimum two hours every time (SL 5).

Public alternatives doesn’t exist, only private ones, and that’s because of unwillingness to include options. The local authority could have hired swimming instructors to cover all instruction in the swimming pools. Then there would no longer be external providers (SI 1).

### 3.2. The Knowledge and Skills of Experts and Non-Experts

#### 3.2.1. Interpretations of Expertise

The SIs described their expertise as being based on long experience working with water. Many had been active swimmers, and some had physical education backgrounds. All SIs stated that they dealt with professional development by attending seminars, courses, reading science, and interacting with other instructors. SI 4 explained that it was a “developing environment” at work and a special “interest in working with swimming.” When talking about aquatic expertise and skills the SIs mentioned the possession of techniques, methods, and tasks, but also personal characteristics such as being patient, fearless, strict, respectful, generous, and kind. Some mentioned the difference between working with smaller groups during regular swimming courses and working with full school classes. Others emphasized the value of daring to work in the water and learning how to use the aquatic environment. In addition, the SIs described their motivation for working in the field of swimming education. 

To me it’s all about getting the children to love being in the water. I spend a lot of time playing and having fun. The children think they only play games and that the lesson is amusing and safe. They don’t recognise that we’re actually practicing water skills (SI 7).

My vision is to teach Norway how to swim. I’m eager to learn new things and ways to teach. This is what I do; I should know it well (SI 2). 

I wish to have the secondary schools as well so that I can follow the same pupils. I’m sceptical about the schools that don’t use me. I hear things from the parents there. Of course, I can’t force them to use me, but I really wished they focused on the same things and not for instance, breaststroke (SI 3).

We have few hours, but we use them to have fun with swimming, games and play, lifesaving and testing. I make the children comfortable in water; it’s a special talent, I think (SI 6).

#### 3.2.2. Teacher Courses

All SIs expected the teachers to have passed a lifesaving test and to have a role in swimming education. However, the SIs described the teacher’s competence as low. Some mentioned apparent low self-esteem and noted that the teachers “don’t know what to do”. Others mentioned different interpretations of roles: “I often see teachers who think they don’t have to be in the water at all” (SI 7); “many teachers think they are off duty” (SI 2); and “that their role is on the bus” (SI 1). All SIs expressed their desire for the teacher to take part in the education to provide guidance, to keep the pupils quiet and focused, and/or teach a smaller group. The SIs used the staff as security, for teaching a group with either low or high abilities, or as assistants when needed. According to the SIs, building teachers’ competence seemed a common solution to improve collaboration and swimming education. Reflections on why competence was low often produced similar answers including “little interest”, “few scheduled hours”, “little experience”, and not “being suited”. Many SIs had doubts about how teachers would manage to perform as swimming educators despite the material they were handed and the experience they gained after participating in swimming classes. Others mentioned that they thought many teachers could manage to deal with it.

Some schools have teachers who are engaged and bother to ask for help. I have experienced teachers who think asking is embarrassing because they are educated teachers. To acknowledge that this is not their usual territory can be hard. They’ll manage to have swimming classes if they bother to take courses. I think it’s a matter of economy and priorities from the schools, but also interest. Once I held a seminar on swimming. Six hundred teachers were present; only four of them participated in my course in the pool (SI 2).

I believe there are many teacher education institutions with little or no swimming education. Some have good offers, but there is no use in good teacher education if you don’t use that specific knowledge. They must participate in courses and refresh their knowledge. They must teach swimming regularly to stay updated (SI 7).

The SLs also reflected upon the awareness of offering courses to their teachers.

I don’t think it’s right to send all general classroom teachers to swimming courses. We need to choose those with both knowledge and an interest for teaching it. I don’t think everyone can be good at it (SL 4).

#### 3.2.3. Professional Development

A good dialogue and a desire for mutual co-operation seemed important to all groups in order to make the outsourcing arrangement successful. All teachers agreed that receiving teacher training and courses would help them be “better prepared”, but the professional benefits of working with an “expert” were also mentioned as valuable:

I pick up things that the instructors do, but if I were on my own, I would have asked for an up-grading course. I think the best solution would be to have a little bit of swimming each year and more swim practice once every year (Teacher 4).

When participating in swimming classes you automatically build your own resource bank. I learn from the instructors all the time. I don’t write anything down, but remember many clever tricks and exercises. With teacher training and courses, I think you are better prepared with even more tools and ideas (Teacher 3).

Although most teachers and SLs were eager to talk about the benefits of outsourcing, one teacher noted his dissatisfaction with the teamwork between him and the external provider:

I miss the professional collaboration with the instructor. I have many questions regarding swimming technique and ways of teaching, but I’m not sure which methods she uses or what knowledge she has. I don’t know how she teaches. I think more dialogue with the teacher would have been great, but I feel it’s not a priority for the school leaders (Teacher 1).

The SL at Teacher 1’s school commented that he thought his teacher collaborated closely with the SI regarding planning and organizing the classes. When reflecting on the hiring process, a number of SLs had little information, while others showed an interest in hiring high-quality SIs.

### 3.3. Experiences That Are Educationally Valuable and Those That Are Not

The SLs described the swimming curriculum aims as “unrealistic”, “high”, “difficult”, and “demanding”, although many were not exactly sure about the concrete aims. All SIs explained that the aims were similar to the learning strategies that they always worked with. Being safe in the water and able to stay afloat were important for all of them. However, most of the SIs felt that swimming a distance was harder for pupils with less water experience. They did necessary adjustments and focused on the sensation of mastering water and learning to enjoy it rather than reporting high-scoring results. Diving, jumping, front crawl and back crawl, lifesaving, games, and play were common areas of content in the classes, often hidden in games.

A notable number of informants were worried that they did not have enough time for the pupils to get sufficient training to reach the aims. Some also admitted that there was no time scheduled for additional swimming after grade four to achieve further progress, or to fulfil the curriculum aims in swimming after grade seven. Some of the SLs had the opportunity to offer a few hours’ extended education. All groups of informants agreed that the schools’ economic situations and access to swimming pools were of major importance in the extent of the swimming education that was provided.

#### Measuring Quality and Results

For SIs, the quality of their work was shown in the pupils’ behavior and skill development. None of the SIs mentioned that SLs checked the quality of their work, but that parents, teachers, and pupils all evaluated their work together. “I do the quality check. School leaders have never visited me. Sometimes parents and other teachers have visited, but never a principal. Out of sight, out of mind” (SI 2). “We only use adults; well-experienced and educated swimming instructors in school swimming. To us, that makes the quality good” (SI 6).

SLs and teachers stated that the external providers’ ability to deliver high-quality education was based on trust. However, one teacher expressed his uncertainty about the instructor’s pedagogical skills. Both groups mentioned information from pupils as valuable in evaluating the quality of the tuition, together with observational notes from the participating teacher. They were also aware that not achieving the curriculum aims indicated that more time was needed for swimming lessons. 

It’s not good enough, because many still don’t know how to swim. Some can swim 200 m, others 25 and the rest can swim less. It’s a security having our own teacher with them who’s educated (SL 1).

What’s important is that the children get an offer that both works, provide results and that the children have fun. Whether it’s a teacher, external instructor or a swimming club instructor, the children are the main focus. A school leader with this in mind hires teachers with the right competence (SI 7).

## 4. Discussion

### 4.1. Teaching Swimming with HSPE Teachers (Internal) or External Providers

The benefits and risks should determine whether to outsource swimming education or not. Common benefits of outsourcing mentioned in the literature include saving costs, reducing staff, freeing teachers to concentrate on core activities, external expertise, flexibility, effectiveness, and high quality of tuition [33]. The results showed that in many cases, outsourcing was a welcome option for the delivery of swimming education in primary schools. The SLs and teachers at schools where outsourcing had been practiced for years seemed content with the arrangement and had no intention of changing it, since they saw it both saving them work and releasing teachers for other subjects. Many also had no competence or knowledge, and little or no experience in organizing swimming education.

The reasons for outsourcing of swimming instruction are similar to those in previous research [29], in terms of the demand for human and physical resources. Outsourcing, however, was not without risk. Commonly mentioned risks in outsourcing services include costs, employee morale problems, over-dependence on a supplier, lost corporate knowledge and future opportunities, and dissatisfied customers. Outsourcing may fail because of inadequate definition of requirements, poor contracts, lack of guidance in planning or managing an outsourcing initiative, or poor supplier relations [34].

#### 4.1.1. Teachers’ Competence

Being educated in physical education does not imply that teachers are confident to deliver swimming education. The SLs had trouble finding staff willing to participate in swimming classes, and this was confirmed by several SIs who observed teachers participating with little interest or engagement. More interestingly, the study gave insight into how teachers were pleased not to have to take swim classes, based on their personal interest. This leads to two major questions: Can one neglect a part of the curriculum on the basis of personal preferences? Why is swimming education a neglected theme in the educational system? 

We know that the availability of swimming education varies in Norwegian institutions that offer teacher trainees specialized higher physical education. The curriculum and course schedules vary from 0–70 hours of swimming related education through the bachelor’s degree between these institutions. This implies that whether teachers are prepared to teach swimming classes depends on the priorities set in the educational institutions. If swimming was of equal importance in teacher education as it is in the primary school curriculum, teachers should be better prepared to lead swimming classes after graduation. Moreover, since we know that many teachers in primary schools have no specialization in physical education, there needs to be an option for attending a swimming educator course as part of the general teacher education program.

#### 4.1.2. Prioritizing

Despite the Government’s attempts to improve swimming education in primary schools with actions such as revising the curriculum, granting resources for more swimming, and publishing supporting material, there is evidence of an ongoing lack of interest and enthusiasm for swimming education. This is shown in the small numbers of scheduled swimming classes and overshadowing by other subjects. The SIs’ impression was that the different interests of the schools determined how much of their resources were spent on different subjects and activities. Our findings show that prioritization varied. Some schools had staff with both the relevant education and knowledge to provide swimming classes, but prioritized cover for other subjects first. There are reasons to believe that the prioritizing of physical education has connections with the national curriculum’s focus on basic skills like reading, oral expression, writing, numeracy, and the use of digital tools [35]. SLs and teachers mentioned Norwegian, Mathematics, English, and Science as receiving more emphasis than more practical subjects such as physical education, arts and crafts, and music. 

An interesting thought is what would happen if physical skills and/or kinaesthetic competences were part of the basic skills stated in the curriculum. Inactivity and obesity among children are a global concern and promoting physical activity is one of the main recommendations to tackle this problem [36]. The trend, which is also emerging in Norway, is a concern for The Norwegian Directorate of Health. It has commented on the significance of successful initiatives and actions against this health threat [37]. This supports the argument that SLs must give higher priority to physical education and swimming, especially with the curriculum demanding that fourth graders should pass a compulsory swimming test.

#### 4.1.3. Organizational Issues

The larger schools had more economic freedom than smaller schools to cover the cost of outsourcing and hiring physical education teachers. For some it was necessary to make changes in the budget, while others did not have control over such costs. Outsourcing was not considered a cheap solution, in contrast to earlier studies [21,23]. However as Griggs [23] states it: “When the opportunity to give up the delivery of physical education to the nearest confident person in a tracksuit arises, primary schools take it, especially when it only costs £20.” According to Belcourt [19], studies show that outsourcing is not as cost-effective as expected in some cases. One could argue that hiring a qualified physical education teacher to cover all aspects of physical education would be a more economical and sustainable solution. There are 2858 primary and secondary schools in Norway, of which 30% have <100 pupils, 40% have 100–299 pupils, and 30% have >300 pupils [38]. There has been an increase in teaching positions at first to fourth grade levels after more resources were granted. Middle- and large-sized schools should therefore have enough pupils and resources to employ a regular swim teacher in a half-time position, or in a full-time position combined with other duties. This could be an alternative option.

The schools that were new to outsourcing and planning swimming education seemed not to have control over the demand. There were expressions of significant frustration with the demands from Government; the most frequent factors mentioned were access to a swimming pool, finding qualified staff, and finding a swimming instructor. Organizing was a larger task than expected. Finding information and help was an issue many of the SLs wished was more publicly organized, despite swimming having been on the curriculum for over 70 years. This provides information on how governmental demands are being interpreted and initiated, and can be used in evaluating how schools organize themselves.

### 4.2. The Knowledge and Skills of Experts and Non-Experts

#### 4.2.1. Interpretations of Expertise

The need for competence and expertise was a common reason for outsourcing swimming education. This leads to the dilemma introduced by Blair and Chapel [39] (p. 488) who noted that when external providers deliver physical education, they must have the “knowledge, skill, understanding and expertise to carry out specified work”. Problematizing how external providers may not have all the skills to provide the curriculum leads to the question: What is a highly qualified swimming instructor? 

The SIs’ focus was on acquiring aquatic skills and promoting enjoyment in the water. SIs described their formal education and experience. Most importantly, they showed passion for the element they worked with. They showed well-established understanding of obstacles to learning, and had tools for handling different situations in the water. They were aware of curriculum aims and implemented them in games and play. The teachers’ attention was often on organizational matters like transportation, locker rooms, and the additional workload, while the SLs also mentioned reporting results, economic factors, and the process of delivering an offer with which pupils and parents were satisfied. This is similar to previous research, where sport coaches were viewed as willing teachers of physical education, something many class teachers were believed not to be [21].

#### 4.2.2. Teacher Courses and Professional Development

As previous studies indicate, teachers consider working with an external provider to be helpful for their professional development [18,22,25,26]. Developing confidence and gaining experience seemed to be a key factor, also indicated by other studies [20,28]. The teachers and SLs were positive about receiving more education, but prioritization and interests were major factors controlling the ability to initiate this. SIs argued that teachers would struggle to acquire the same level of competence and training despite courses and professional development. There is a need for continued professional development for teachers attending swim courses. Options could be teachers collaborating, smaller classes, more hours in which to teach swimming, and outsourcing until teachers feel confident about taking over the lessons. A number of teachers and SLs acknowledged the fact that some teachers did not have the skills required to become a good swim educator. However, teachers questioning the SIs’ methods indicates that there are quality differences in external providers as well. This might also be reflected in the pedagogical competences of the SIs. While 100% of the teachers had formal education in physical education, only 43% of the SIs had this formal education. While the Norwegian Swimming Federation offers 36 hours of swimming education to SIs [40], only some of these hours are allocated to pedagogical development. It could therefore be argued that SIs are specialists in the methods used for teaching children how to swim, and the educated teachers are the pedagogical specialists with unique knowledge of each individual pupil and how they respond to different teaching methods. This was also shown in the statement of Teacher 1 who experienced a professional deficit in the SI, a low willingness to communicate, which might show that the pedagogical demands of teaching are not equally considered when hiring external providers. This would argue for a closer collaboration between the SIs and teachers for transferring knowledge not only on the methods for teaching pupils how to swim, but also for the pedagogical skills needed in order to enhance the individual learning of each pupil.

### 4.3. Experiences That Are Educationally Valuable and Those That Are Not

#### Controlling Results and Quality

“Externally provided physical activity programmes, in which physical education specialists provide students with specialist instruction, are a recognised method to avoid relying on generalist teachers in primary school settings” [22] (p. 73). The outsourcing arrangement seemed to some SLs and SIs a convenient “solution” to a “problem” [26]. When swimming education was outsourced, it seemed that the quality was based on a relationship of trust. “Failure to manage outsourcing relationships properly, perhaps through service level agreements, may reduce customer service, levels of control and contact with customers” [41] (p. 839). In a number of cases, the teachers who were participating together with an external provider were the only ones at the school who had information about the content. SIs believed that once the school outsourced, they let go of all responsibility. The fact that the staff collaborating with instructors had both varied swimming competence and different interpretations of their roles during the classes made quality measurement demanding. The SIs’ reflection of SLs trusting them to deliver the curriculum was confirmed by the SLs stating how they trusted “professionals” and “experts” to do their job, as found in previous studies [26,29]. This however, can be a concern for inattentive teachers and SLs.

The SIs were conscious of the need to deliver high-quality classes. They also noted that pupils’ feedback of “having fun” could be misleading and might not provide reassurance that the classes provided high-quality swimming education. Discussion evolved around which pedagogical platform SIs use. A physical education teacher is bound by the regulations of the school. External providers are outside of the schools’ regular quality control processes, hence SIs in a municipality outsourcing arrangement work under the same regulations as other municipality employees. SIs with an educational background from the swimming federation shared a similar background with a basis in the curriculum aims, which the swimming federation helped to develop. All SIs interviewed had formal swimming education. However, what can and should be questioned is what is being operationalized when schools outsource to private providers without any formal education. This can be one of the risks when outsourcing educational services.

An obvious risk for the external providers in delivering low-quality classes is that pupils do not reach the curriculum aims and that their contract with the school is not renewed. A concerning finding was that many pupils did not meet the aims and the result was explained as being due to too little time to practice. The SIs also described how they implemented self-rescue as a theme in the classes, but often did not have time to cover extended lifesaving education. This theme was not widely mentioned by SLs and teachers, showing poor knowledge of both the content of the classes and the exact learning outcomes. This can be connected to the prioritization of physical education and subject content.

Some limitations were present in the study. One can argue that to answer the research question, informants from the other side should also be included, these being teachers and SLs who did not participate in any outsourcing arrangements. There is reason to believe that the ideal model would have an educated teacher, with high swimming competence, conducting comprehensive swimming education in every school. Including this group may have identified other perspectives on why outsourcing was not used and how the teachers delivered swimming education. Including this group may also have implied the availability of resources such as access to swimming pools, economic resources, and schools offering full-time positions for specialist physical education teachers. The reason for not including this group of informants was that our primary interest was in outsourcing arrangements, without comparison to other more common forms of delivering physical education. However, we acknowledge that this is a limitation to our study and should be the aim of a future study.

The reason for interviewing three different groups was our interest in different perspectives relating to different outsourcing solutions. There is also the possibility that obtaining pupils’ views as part of the data could have added information about the learning outcomes. However, the age of the pupils and the basis of the study did not align with this. This would be an interesting topic for a future study integrated with the pupil’s enjoyment in the swimming classroom, as this is an important element of physical education and learning how to swim.

## 5. Conclusions

This study aimed to explore different mechanisms and consequences relating to the use of external providers in swimming education for Norwegian primary schools. However, although these results are related to Norway, they may also be helpful for other countries with compulsory swimming education in schools or trying to implement this as a part of the core curriculum. A key element in the study was to investigate ideas about outsourcing arrangements from the point of view of SLs, teachers, and SIs. It was anticipated that all would aim at the same goal: Pupils achieving the curriculum aims. The results showed that outsourcing was a functional option. It was viewed as an easy way to make sure that swimming education was delivered. The pupils received better education than the teachers would have managed on their own. To some SLs, it was timesaving and safe. The SIs were passionate about their work and their statements underpin the purpose of physical education: To inspire lifelong enjoyment of being physically active.

The results indicated that outsourcing was a solution for schools when:

- They had no competence or knowledge relating to organizing swimming education.
- They had little or no experience in organizing swimming education.
- They wanted inspiration, professional development and experience related to swimming education.

When outsourcing, there is a need for a change in the way SLs think of:

- Participating staff—are they qualified?
- Participating staff—what role do they play in the classes?
- Measuring quality—do we have the right tools?
- Prioritization—are adequate resources spent on ensuring that the pupils reach the curriculum aims?

If the school has qualified staff, one option is to evaluate how this competence can be used before engaging in outsourcing. The different answers with regard to how teachers and participating staff interacted with the SIs posed questions on how we can trust people to properly fulfil their contracted roles. Communication appeared to be a solution for improving collaboration across sectors, along with more knowledge. We argue that there are many ways to gain competence in swimming education and a combination of these may be appropriate. Professional development through participation with external providers may be of great value if the collaboration between the parties is good. There is an equal responsibility in sharing knowledge and being open to receiving it. More teachers participating in courses may also make both competence and professional development more available inside the school. Educational support material available online should also be a source of knowledge.

At a higher level, we suggest that physical education should be included as one of the basic skills (parallel to mathematics, reading, writing, oral communication, and digital skills) in order to raise the status and prioritization of the subject. Alongside this we suggest a change in teacher education, implementing more swimming education to prepare future teachers to engage in swimming education with knowledge, interest, and enthusiasm.

## Figures and Tables

**Table 1:** Definition of kinematical variables chosen for analysis.

Group	Swimming Instructor	School Leader	Physical Education Teacher	Total
Private	3	3	1	5
Swimming club	2	2	2	6
Municipality	2	2	1	6
Total	7	7	4	18

## Data Availability

The data presented in this study are available in the article.
